# Is a dissonance-based group intervention targeting thin-ideal internalization a successful potential add-on for specialized eating disorder care? A randomized feasibility and acceptability pilot study

**DOI:** 10.1186/s40337-023-00784-1

**Published:** 2023-05-02

**Authors:** Joyce Maas, Mladena Simeunovic-Ostojic, Nynke M. G. Bodde

**Affiliations:** 1 Centre for Eating Disorders, GGZ Oost-Brabant, Wesselmanlaan 25a, 5707 HA Helmond, The Netherlands; 2 Department of Medical and Clinical Psychology, Tilburg University, Warandelaan 2, P.O. Box 90153, 5000 LE Tilburg, The Netherlands

**Keywords:** Eating disorders, Cognitive dissonance, The body project, Feasibility pilot, Specialized eating disorder care

## Abstract

**Background:**

Dissonance-based eating disorder programs have successfully targeted body dissatisfaction by challenging the thin beauty ideal in the preventive context and in groups of patients with a subthreshold and full threshold DSM-5 eating disorder. As there is a need for interventions specifically targeting thin-ideal internalization in (highly) specialized treatment centres, the present study adapted Stice’s et al.’s Body Project for its use as an add-on treatment for severe eating disorders with the aims to identify whether it was feasible and acceptable in this treatment context, to determine any necessary modifications with regard to the treatment and study procedures, and to test preliminary effectiveness.

**Methods:**

The study was a randomized controlled pilot/feasibility trial. Thirty patients started in the Body Project group and 25 in the Psycho-education group. Measurements took place pre- and post-intervention, and at three and six months follow-up. Patients and staff evaluated treatment and study procedures, and patients completed questionnaires on thin-ideal internalization, body dissatisfaction, self-objectification, negative affect and eating disorder pathology.

**Results:**

The Body Project group and Psycho-education group both proved highly feasible and acceptable, as well as preliminarily effective, based on quantitative scores and qualitative feedback. Preliminary analyses showed that treatment effects did not differ between treatment groups. As both groups were an add-on to standard treatment, treatment effects cannot be disentangled from effects resulting from standard treatment. Qualitative feedback for the Body Project group included several recommendations for future implementation: increasing the number of treatment sessions, creating homogeneous therapy groups, and optimizing timing of the treatment.

**Conclusions:**

Future research should examine further modifications to the Body Project group for severe eating disorders, as well as for whom, and when in the course of treatment the intervention is most effective. The present study also showed the benefits of implementing a structured Psycho-education group.

**Plain English summary:**

We tested the feasibility and acceptability of a group intervention targeting the thin beauty ideal (Body Project group) in patients with severe eating disorders and compared this intervention to a group intervention focusing on psycho-education about eating disorders (Psycho-education group). Both interventions were added to standard treatment. We adapted the protocol for patients with severe eating disorders. Both the Body Project group and the Psycho-education group were evaluated by patients as well as staff as highly feasible and acceptable, and effects were positive. Treatment effects did not differ between treatment groups. As both treatments were an add-on to standard treatment, treatment effects cannot be disentangled from effects resulting from standard treatment. The study suggested further modifications to the Body Project group. Future research should examine these modifications as well as for whom, and when in the course of treatment the intervention is most effective. The present study also showed the benefits of implementing a structured Psycho-education group.

## Background

Body dissatisfaction is a major predictor of relapse in patients treated for anorexia nervosa and bulimia nervosa [1, 14]. According to Stice’s dual pathway model [27], thin-ideal internalization produces body dissatisfaction, which in turn leads to restraint eating and negative affect, increasing the risk for the onset of eating disordered pathology [28, 33, 35]. Thin-ideal internalization, or the extent to which an individual cognitively ‘buys into’ socially defined ideals of attractiveness and engages in behaviours designed to produce an approximation of these ideals [38], is a result of pressures to attain the excessively thin female body standard placed on individuals by the media, but also by family, peers, school subcultures and interpersonal encounters [34]. Stice’s model has received great empirical support (e.g., [2, 5, 12, 24]).

Originally developed in the context of eating disorder prevention, the Body Project [32], a dissonance-based eating disorder program, targets body dissatisfaction by challenging the thin beauty ideal. A great deal of evidence supports the Body Project as an effective preventive treatment for eating disorders. It uses cognitive dissonance to address thin-ideal internalization, body dissatisfaction, and eating disorder symptoms. The theory of cognitive dissonance proposes that when there is an inconsistency between an individual’s beliefs and behaviours, the resulting discomfort will motivate them to change their attitude or behaviours to reduce this inconsistency [9]. More specifically, in the Body Project women with body image concerns voluntarily and actively engage in verbal, written and behavioural exercises in which they challenge beliefs about the thin-ideal. For example, participants write and present to the group problems associated with the thin-ideal and the individual and societal costs of attempting to attain the thin-ideal. The cognitive dissonance and associated discomfort then motivates the individual to alter their thin-ideal internalization, in turn reducing body dissatisfaction, negative affect, restraint eating, and eating disorder symptoms.

A meta-analysis by Stice and colleagues [28] concludes that the Body Project produced larger reductions in thin-ideal internalization, body dissatisfaction, negative affect, restraint eating, and eating disorder symptoms than minimal-intervention control conditions, but also when compared to alternative interventions. Effects were strongest for thin-ideal internalization, in line with the intervention’s goal. Stice et al. [29, 31, 36 ]furthermore showed that the program produced relatively similar effects between different racial/ethnic groups.

Changes are likely not limited to the explicit behavioural level. Kant et al. [13] showed that the Body Project was able to change implicit attitudes, as measured with an Implicit Association Test. Interestingly, Stice, Yokum and colleagues [28] used fMRI and found greater reductions in responsivity of regions involved in reward valuation to thin models.

Several trials also have shown support for the use of a modified version of the original Body Project prevention program as a group treatment for women with a subthreshold and full threshold DSM-5 eating disorder [30]. In this modified program, referred to as the Body Project Treatment, Stice et al. developed new exercises to address body dissatisfaction, thin-ideal internalization, as well as eating disorder symptoms. Several trials have provided support for the Body Project Treatment for its use across different eating disorder types [28–31, 33, 35, 36].

The evidence for the Body Project programs in the preventive context and in groups of patients with a subthreshold and full threshold DSM-5 eating disorder is promising so far. However, this finding requires confirmation in patients seeking treatment in (highly) specialized eating disorder services. Specialized (second-line/secondary care) and highly specialized (third-line/tertiary care) treatment centres likely include a different and more severe subset of patients than those included in previous trials investigating the Body project programs. The majority of patients with eating disorders in the Netherlands are referred by their general practitioner to first-line general psychiatry mental health care or second-line specialized care services for eating disorders, where the stepped care approach and a first- or best-choice treatment can be provided. Third-line mental health care for eating disorders (highly specialized centres, tertiary care) in the Netherlands treats patients that were not (or would not be) treated successfully in first-line or second-line specialized care services. This includes patients with a duration of illness ≥ 2 years, an eating disorder with a high severity level (e.g., extremely low BMI [< 15], highly restrictive or chaotic eating pattern), treatment-interfering psychiatric comorbidity and/or somatic comorbidity and/or two or more comorbid Axis-I or Axis-II disorders and psychosocial dysfunction. Our highly specialized treatment centre for eating disorders, Centre for Eating Disorders (GGZ Oost Brabant, Helmond, the Netherlands), is a supra-regional treatment centre that provides secondary mental health care services in our own region of the country, as well as high-intensity and highly specialized matched care treatment programs to patients with severe and complex eating disorders, who have often received a number of ineffective and/or insufficient treatments before they are referred to us.

Patients in treatment at our centre expressed the need for an intervention targeting the thin-ideal, which instigated setting up the present study. Typical for (highly) specialized eating disorder centres, our treatment is multimodal. The Body Project was therefore tested as an add-on to treatment as usual. We chose to add the Body Project instead of the Body Project Treatment, as treatment as usual already includes many treatment modules aimed at eating disorder symptoms, but lacks interventions targeting the thin beauty ideal. Treatment as usual starts with motivational interviewing and establishing a regular eating pattern. More specifically, the present study was set up to test the feasibility and acceptability of the Body Project in a highly specialized care setting in third-line mental health care treatment. Given the complexity of the patient group in this treatment setting as well as the infrastructure within and across (highly) specialized treatment centres for eating disorders, important questions concern the feasibility and acceptability of the Body Project. Feasibility and acceptability meant testing both the delivery of the groups as well as testing the study procedures from the therapists’ and team’s perspective, as well as the satisfaction of patients with regard to the groups and study procedures. Following recommendations for these types of studies (e.g., [16, 17, 21, 26, 37], the present study used quantitative and qualitative methods to study the intervention as well as the study design, and to prepare the treatment centre and team for a future larger-scale study. Under this definition, the primary role of the present was not to test effectiveness of the intervention, nor to obtain information about effect sizes with any certainty. We did investigate the likely effects of the interventions, but, In line with the recommendations of Lancaster et al., and Thabane et al., we clearly labelled these tests as preliminary tests.

Patients were randomized to receive either the Body Project group or a Psycho-education group, added on to standard multimodal treatment. The three main objectives of the study were therefore to identify whether (1) the Body Project and Psycho-education groups are feasible and acceptable in a highly specialized treatment centre for eating disorders, (2) to determine any necessary modifications with regard to the treatment and study procedures for this specific group, and (3) to test preliminary effectiveness. Patients as well as staff evaluated treatment and study procedures, and patients completed questionnaires on thin-ideal internalization, body dissatisfaction, self-objectification, negative affect and eating disorder pathology to preliminary investigate effectiveness of the groups.

## Material and methods

### Participants and procedure

The study was a randomized controlled pilot/feasibility trial with measurements pre- and post-intervention, and at three and six months follow-up. One-hundred-sixty-one in- and outpatients (15–65 years) with anorexia nervosa or bulimia nervosa, following regular treatment for their eating disorder at the Centre for Eating Disorders, were assessed for eligibility. Patients had to be in treatment for at least 10 weeks to first get motivational pre-treatment and to establish a pattern of regular eating. Exclusion criteria were being male, a diagnosis of schizophrenia or other psychotic disorders, substance abuse, medical complications that may hamper the interpretation of results (medical conditions that causes weight changes), and/or changes in dose or type of psychiatric medication in the previous four weeks. As we offer specialized eating disorder care in third-line mental health care, our patient population consists mostly of patients with severe eating disorders and/or (multi)comorbidity (e.g., depressive disorder, obsessive–compulsive disorder, personality disorders, somatic comorbidity, including those resulting from the eating disorder and/or severe underweight), who did or would not respond adequately to first-line or second-line treatment for eating disorders and/or need a more intensive level of care. As the aim of the study was to assess feasibility and acceptability in this type of patient population, we did not exclude participants with a severe eating disorder or comorbidity (other than the previously mentioned schizophrenia or other psychotic disorders and substance abuse).

Eligible patients were given the patient information letter and the informed consent form, as well as a brochure explaining the law and the rights of patients participating in medical research. Patients were asked to make their decision within two weeks. The study was approved by the medical ethical committee Oost-Nederland (East-Netherlands).

**Fig. 1:**
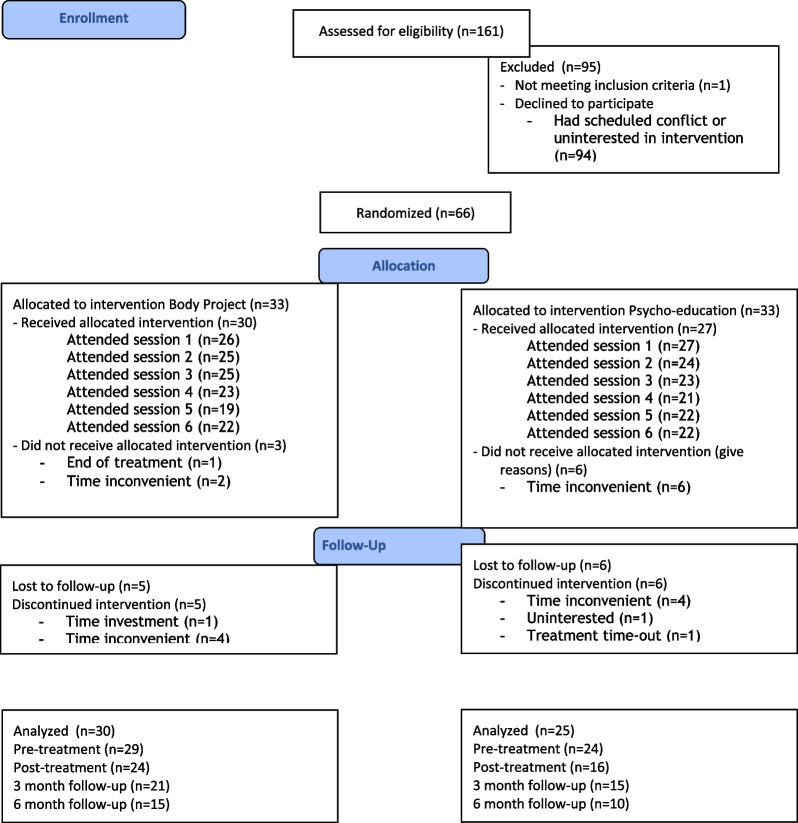
Patient flow-chart

Patients who gave informed consent were randomized to the Body Project group or the Psycho-education group. Random assignment was based on an adaptive stratification procedure to control for possible effects of illness duration (< 5, or 5 + years) and eating disorder subtype. Thirty patients started in the Body Project group and 25 started in the Psycho-education group (see Fig. 1 for the patient flowchart). Fourty-nine percent suffered from a comorbid condition: depressive disorder (*n* = 12), personality disorder (*n* = 11), anxiety disorder (*n* = 7), autism spectrum disorder (*n* = 3), attention deficit hyperactivity disorder (*n* = 2), other (*n* = 4). Fourty-four percent were on medication. Patient characteristics are presented in Table 1. All between group differences were non-significant (all *p*’s > 0.14).

**Table 1:** Patient characteristics

	Body project (*n* = 30)	Psycho-education (*n* = 25)
Age	*M* = 31.13, *SD* = 13.49	*M* = 29.96, *SD* = 10.00
BMI	*M* = 20.55, *SD* = 7.56 AN = 16.49, *SD* = 1.98 BN = 24.97, *SD* = 3.32	*M* = 19.46, *SD* = 8.24 AN = 16.67, *SD* = 2.52 BN = 25.35, *SD* = 7.04
Diagnosis	AN = 20, BN = 10	AN = 21, BN = 4
Duration	< 5 = 11, 5–10 = 15, > 5 = 5	< 5 = 8, 5–10 = 12, > 5 = 5
Comorbidity	Yes = 16	Yes = 12
Medication use	Yes = 12	Yes = 12

Both interventions consisted of six weekly one-hour group sessions and were an add-on to regular treatment. Regular treatment starts with motivational interviewing and consists of common multimodal inpatient and day clinic modules that are based on a comprehensive system designed to normalize eating patterns, stabilize or increase weight, and eliminate compensatory behaviours. The theoretical orientation of the program is based on Enhanced Cognitive Behaviour Therapy, Specialist Supportive Clinical Management, and the Maudsley Model for Anorexia Nervosa Treatment for Adults (MANTRA). Patients receive different treatment modules during the day with supervised or unsupervised snacks/lunch in between. Treatment modules are nutrition management (including regular weighing, food diaries, and fear food exposure), perfectionism, self-compassion, Cognitive Remediation Therapy (CRT), Competitive Memory Training (COMET) for self-esteem, psychomotor therapy, drama therapy, and cognitive behaviour therapy. The treatment also includes medical assessments, weekly group psychotherapy, individual psychotherapy, and family interventions. The treatment team is multi-disciplinary, including psychiatrists, psychologists, nurses, dieticians, social workers, psychomotor, and drama therapists.

Patients usually have therapy sessions from 9 AM to 3 PM and receive treatment for several months, typically 12–18 months in total. Patients with bulimia nervosa receive treatment two days a week, patients with anorexia nervosa three to five days a week. Patients needing inpatient treatment stay at the treatment centre 24/7, for a maximum of 12 weeks, and then continue to day clinic treatment or treatment elsewhere.

### Body project group

The Body Project group was based on a detailed manual of the six-session Enhanced-Dissonance Body Project [32]. The intervention includes six weekly one-hour group sessions and is designed to induce cognitive dissonance regarding the pursuit of the thin-ideal. In this program, patients with body image concerns voluntarily and actively engage in verbal, written and behavioural exercises in which they critique the thin-ideal, for example critiquing the thin ideal in group discussions, arguing the pursuit of the thin ideal in role-plays, writing letters about (the costs of) the thin-ideal, acknowledging positive personal physical features while looking in a mirror, engaging in body activism, and generating verbal “quick comebacks” to thin-ideal statements. We translated the manual to Dutch and made several changes to allow for a better fit with our patient group, as the original manual is targeted at students and/oroutpatients. For example, in the self-affirmation exercise, the original protocol asks to wear as little clothing as possible. As wearing little clothing was considered too triggering, we omitted this instruction. For a complete overview of all the interventions and changes to the original program, see Appendix 1. The Body Project group was led by a qualified psychologist and a nurse/counsellor.

### Psycho-education group

The Psycho-education group also involved six weekly one-hour group sessions. It included topics that are of importance to eating disorder patients. The following themes were discussed: the background (definition, symptoms, diagnosis, biopsychosocial model) and consequences (complications) of eating disorders, the Minnesota starvation experiment, body image, normal eating, self-monitoring, and the physiology behind eating, biopsychosocial risk factors, the ineffectiveness and adverse effects of compensatory behaviour, motivational stages, psychological treatment and medication options, as well as how family/partner/friends can be involved in the therapy process. The Psycho-education group was led by a qualified specialist nurse and psychologist (a different psychologist than the one leading the Body Project group).

## Measures

### Feasibility and acceptability—evaluation of treatment and study procedure

#### Patients

The patient evaluation questionnaire contained multiple-choice items with a five-point scale, including the option to add comments regarding perceived strengths, weaknesses, and feasibility of the intervention. The questionnaire was divided into five sections, each exploring different aspects of the treatment and the trial process: (i) general assessment of the treatment (ii), the study procedures, (iii) the effectiveness of and relationship to the therapists and group members, (iv) the perception of change in relation to feelings about self and body, the eating disorder and life in general, and (v) the acceptability of the treatment. At the pre-treatment assessment patients’ expectancy with regard to treatment and readiness for change was also assessed.

#### Therapists

After each session both therapists who delivered the treatments completed a short checklist evaluating the session. Therapists also tracked each patient’s attendance, participation, and homework completion level, and completed an evaluation questionnaire at the end of the study, including questions about the therapy manual, the intervention delivery and the trial procedures. Willingness to offer the treatment in the future was also assessed.

#### Treatment directors and team

Treatment directors completed an evaluation questionnaire that included questions about study procedures as well as noticeable effects (positive or negative) on participating patients’ symptoms.

### Preliminary effects – questionnaires

We used the following questionnaires to assess preliminary effectiveness.

#### Anxiety

The Beck Anxiety Inventory (BAI; [3] is the most widely used instrument for assessing anxiety symptoms. It is a self-report measure that reliably differentiates anxious from non-anxious groups in a variety of clinical populations, and discriminates anxiety from depression. The scale consists of 21 items, including physiological and cognitive features of anxiety. Participants are asked to rate how much they have been bothered by each symptom over the past week on a 4-point scale ranging from 0 to 3, with higher scores reflecting more symptoms. Cronbach’s alpha in the current study was 0.86.

#### Depression

The Beck Depression Inventory (BDI-II; [4] is among the most widely used scales for self-ratings of depressive symptoms. It contains 21 items, with item scores ranging from 0 to 3, which are added to obtain a composite score. Higher scores mean more depressive symptoms. Cronbach’s alpha in the current study was 0.88.

#### Negative affect

The Positive And Negative Affect Scale (PANAS; [41] was used to assess negative affect. As we were only interested in negative affect, patients were only asked to answer the extent (on a five-point scale with 1 meaning hardly/not at all and 5 meaning strongly) to which they experienced the negative emotions contained in this questionnaire, which is a common procedure. The negative scale possesses strong convergent and discriminant validity, and has also shown to be reliable [40]. In the current study, Cronbach’s alpha was 0.91.

#### Eating disorder symptoms

The EDE-Q [19] is a 36-item self-report scale that focuses on the previous 28 days to assess key behavioural and attitudinal features of eating disorders and the severity of the psychopathology of eating disorders. It consists of four subscales measuring four eating attitudes: Restraint, Eating Concerns, Shape Concerns, and Weight Concerns. A higher score indicates a higher level of eating disorder psychopathology. Cronbach’s alphas were 0.79 for Restraint, 0.70 for Eating Concerns, 0.90 for Shape Concerns, 0.73 for Weight Concerns, and 0.93 for the total score.

#### Body dissatisfaction

The Body Attitude Test (BAT; in Dutch Lichaams Attitude Vragenlijst; [23]) consists of 20 items rated on a six-point scale (0–5). It was developed to assess body attitudes and subjective body experience of patients with eating disorders. The maximum score is 100 and a cut-off score of 35 is suggested by Probst et al. The higher the score, the more disturbed the body experience. Factor analysis identifies four factors: the negative appreciation of body size, lack of familiarity with one’s body, general body dissatisfaction, and a rest factor. Various studies demonstrated the validity and reliability of the BAT. Cronbach’s alphas in the current study were 0.72 for the negative appreciation of body size, 0.78 for lack of familiarity with one’s body, and 0.69 for general body dissatisfaction.

#### Body objectification

The Objectified Body Consciousness Scale (OBCS; [20], a measure of self-objectification, is comprised of three subscales: Surveillance, Body Shame, and Control Beliefs. The Surveillance scale measures the degree to which individuals view their physical body from a third-person perspective. The Body Shame scale measures the degree to which individuals ascribe to cultural body standards and feel ashamed if they do not meet these standards. The Control Beliefs scale measures the degree to which individuals believe they can control their weight and shape. Each subscale has 8 items that are rated on a seven-point scale from 1 (strongly disagree) to 7 (strongly agree). Fourteen of the items are reverse-scored. Higher scores correspond with higher levels of surveillance, body shame, and control beliefs. The OBCS has demonstrated high internal reliability and good construct validity [20]. In the current study Cronbach’s alphas were 0.85 for Surveillance, 0.81 for Body Shame, 0.58 for Control Beliefs, and 0.82 for the total score.

The Self-Objectification Questionnaire [10] assesses the degree to which various physical qualities and attributes are valued as part of a woman's physical self-concept. The SOQ asks participants to rank order 10 body attributes, rating how important they believe each attribute to be (0 = least important, 9 = most important). Five of the attributes are appearance-based (e.g., weight, physical attractiveness) and five are competency-based (e.g., physical coordination, physical fitness level). Originally, a total for this scale is computed by summing the total of the ranks for the five appearance-based attributes and subtracting the sum total of the ranks for the five competency-based attributes. However, both appearance-based and competency-based subscales were not reliable. In this study we therefore only present the score for patients’ score on the attribute ‘weight’.

#### Thin-deal internalization

Thin-ideal internalization was measured using Sociocultural Attitudes Towards Appearance Scale-3 (SATAQ-3; [39]. The SATAQ-3 is composed of 30 statements which individuals are instructed to rate on a five-point scale from 1 (definitely disagree) to 5 (definitely agree). The SATAQ-3 yields five subscales: (1) Information – which measures the degree to which an individual acknowledges that TV, magazines, advertisements, and celebrities offer information about what is attractive, (2) Internalization-Athlete – which measures how strongly an individual ascribes to athletic-looking standards of attractiveness, (3) Internalization-General – which measures how strongly an individual ascribes to thinness as the standard for attractiveness, (4) Internalization-Total – which is the summed score of the Internalization-General and Internalization-Athlete subscales, and (5) Pressures – which measures to what degree an individual feels pressured to embody these standards of attractiveness. Eight scores are reverse-scored. Higher scores correspond with stronger thin-deal internalization. The subscales of the SATAQ-3 have demonstrated excellent convergent validity with measures of body image dissatisfaction and disturbed eating [39]. Cronbach’s alphas were 0.93 for Information, 0.81 for Internalization-Athlete, 0.94 for Internalization-General, 0.96 for Internalization-Total, and 0.93 for Pressures.

### Analyses

As subscales of questionnaires were highly correlated, we only presented the total scores in the Results section. A correlation table with correlations between all (sub)scales is presented in Appendix 2.

Next to descriptive statistics to assess acceptability and feasibility, we used multilevel analyses (generalized mixed models) with IBM SPSS version 26 to analyze the data (intent-to-treat), because, in contrast to the pairwise deletion procedure applied by Repeated Measures ANOVAs, multilevel analysis is a powerful approach to treating missing data [22]. Please note that the study was set up as a feasibility and acceptability pilot study, and was underpowered to assess treatment effects. Tests therefore only preliminary investigate effectiveness of the treatments. To make the model more parsimonious and thereby increase statistical power, we reduced the number of parameters by critically looking at the covariance structure. The random intercept model fitted the covariance structure of the dependent variables well and was therefore chosen for all analyses. For every dependent variable separately, we first looked at model including the main effects of group, time, and the group*time interaction. As the interaction effect was never significant, the interaction effect was dropped in the final model of interpretation. We also checked for linearity of time by comparing the model with time as a continuous variable versus the model with time as a discrete variable. As the models did not differ significantly, we chose to add time as a continuous variable, as this was the most parsimonious model.

To control for multiple testing, a significance level of 0.05/9 = 0.005 was used. Within-group effect sizes (Cohen’s d) were calculated in order to evaluate effect sizes of treatment for the Body Project group and Psycho-education group separately. Effect sizes for the time*group interactions were estimated by carrying out Repeated Measures ANOVAs for each of the questionnaire scores (pre- to post-treatment).

## Results

### Attendance, participation, and homework completion

The flowchart in Fig. 1 shows attendance to each session and study drop-out. In the Body Project group 73.33% (84.62% when corrected for initial no-show) of patients attended all sessions, in the Psycho-education group 81.48% attended all sessions.

Patients in the Body Project group scored 1.78 on involvement, and 1.65 on homework completion, which were both scored on a scale from 0 to 2. In the Psycho-education group, patients scored 1.79 on involvement and 1.82 on homework completion.

### Evaluation of the treatments and study procedures

#### Patients

The Body Project group was rated positively by the patients, as was the Psycho-education group. On a 5-point rating scale, with 5 being the most positive score for most questions (see Table 2), patients’ scores on ‘general assessment of the treatment’ ranged from 3.04 to 3.96 (session length and number of sessions were scored 2.96 and 2.64, respectively, with 3 being the most optimal score). For the Psycho-education group, patients’ scores were similar and ranged from 3.35 to 4.06, with scores of 3.13 and 2.88 for session length and number of sessions. Scores on the study procedures ranged from 3.55 to 4.00 for the Body Project group and from 2.94 to 4.18 for the Psycho-education group (and 2.61 for time spent on questionnaires [Psycho-education group: 2.71], with 3 being the most optimal score), scores on ‘the effectiveness of and relationship to the therapists and group members’ ranged from 4.26 to 4.87 in the Body Project group and from 4.24 to 5.00 in the Psycho-education group. Scores on ‘the perception of change in relation to feelings about self and body, the eating disorder and life in general’ (i.e., subjective perception of therapeutic effects) ranged from 2.70 to 3.13 in the Body Project group and from 2.12 to 2.82 in the Psycho-education group, and on ‘the acceptability of the treatment’ scores ranged from 3.04 to 4.14 in the Body Project group and from 3.41 to 4.35 in the Psycho-education group.

**Table 2:** Patient evaluation of the therapy groups and study procedures

Scale	Question 1–5 scale, with 5 being the most optimal score for most questions, exceptions are explicitly mentioned below	Body project mean (sd)	Psycho-education mean (sd)
General assessment of the treatment	General satisfaction	3.61 (0.78)	4.06 (0.75)
	Match with expectancies	3.22 (0.90)	3.35 (0.78)
	Recommendation of the module to others	3.96 (1.02)	4.47 (0.72)
	Session length (1 = too short, 5 = too long)	2.96 (0.64)	3.13 (0.50)
	Number of sessions	2.64 (0.79)	2.88 (0.49)
	Balance between exercises and discussion (1 = too many exercises, 5 = too much discussion)	3.04 (0.47)	3.41 (0.51)
	Homework content	3.70 (0.82)	3.71 (0.69)
	Homework quantity		
	Hand-outs used during sessions	3.57 (0.66)	4.12 (0.60)
Study procedure	Patient information letter	4.00 (0.98)	4.18 (1.07)
	Randomization procedure	3.55 (0.91)	2.94 (0.77)
	Questionnaires – emotional strain (5 = not at all)	3.74 (1.10)	3.82 (0.95)
	Questionnaires – time (1 = a lot of time, 5 = not much time)	2.61 (0.94)	2.71 (0.99)
Effectiveness of and relationship to the therapists and group members	Therapists—clarity	4.87 (0.34)	4.76 (0.44)
	Therapists—persuasion	4.65 (0.65)	4.76 (0.44)
	Therapists—credibility	4.57 (0.59)	4.82 (0.39)
	Therapists – feeling heard	4.74 (0.62)	4.82 (0.39)
	Group—collaboration	4.26 (0.54)	4.53 (0.62)
	Group—atmosphere	4.39 (0.58)	4.24 (0.75)
	Respectful treatment	4.87 (0.34)	5.00 (0.00)
Positive perception of change	Body image	2.91 (1.20)	2.12 (1.32)
	Eating disorder	2.70 (1.46)	2.65 (1.37)
	Self-esteem	3.09 (1.12)	2.53 (1.28)
	Enduring changes	3.13 (1.22)	2.82 (1.47)
Acceptability	Useful	3.96 (1.11)	4.35 (1.06)
	Personal fit	3.83 (1.37)	4.12 (1.11)
	Strain (1 = a lot, 5 = not at all)	3.04 (0.93)	3.41 (1.00)
	Discomfort	3.48 (0.85)	3.59 (1.00)
	Negative effects (1 = a lot, 5 = not at all)	4.14 (0.97)	4.12 (0.93)

Patients were positive about the Body Project group and commented that they hoped for the treatment to be included in the standard treatment package. Several patients mentioned the Body Project group was confronting or ‘out of there comfort zone’, but also mentioned this to be important in order to induce change. Most patients also mentioned that a program consisting of six sessions was too short; they needed more practice in order to induce long-term change. One patient mentioned that the program especially changed how she looked at other peoples’ bodies. One patient did not feel comfortable in the group, as she was an older woman with a severe and enduring eating disorder, whereas the other patients in the group were much younger. She suggested more homogenous groups in the future.

Patients were also very positive about the Psycho-education group. Although psycho-education is already part of our standard treatment, it is rather incorporated in different treatment modules instead of it being offered as a separate group treatment. Patients commented that they liked the group format and that the repetition of the information that they have heard before in treatment was helpful. Several patients mentioned the group gave them an opportunity to discuss the information more than in regular treatment, which was viewed as helpful, as information about Minnesota starvation experiment and normal eating were viewed as triggering by these patients.

In the evaluation questionnaire, patients were also asked whether they had a preference for one of the two treatment groups before randomization and whether this preference had changed. Four patients who were randomized to the Body Project group answered they had a preference for the Psycho-education group before randomization, of which one changed her mind after completing the group. This means that all but these three patients were satisfied with the result of randomization.

#### Expectancy and readiness for change

Patients in the Body Project group scored 68% on the amount of faith (0–100%) they had in their ability to fight the eating disorder before the start of the group. After the group their score was 66%. In the Psycho-education group this score changed from 62 to 71%. Please note that both groups were only a small part of an extensive treatment (60 min for 6 weeks in a 12–18 month treatment with therapy sessions 2–5 days a week), and patients were already in treatment before the start of the study.

#### Therapists

Table 3 shows means and standard deviations of the post-session therapist checklists that were completed after each session. Scores were generally very positive. That is, most items on the checklist scored > 4 (5 was the maximum score). Preparation (reading the session protocol and preparing the session with the co-therapist) scored < 4, for both groups (2.45 to 3.47). In the Psycho-education group using appropriate humor and involving quite patients also scored < 4 (3.40 and 3.83, respectively).

**Table 3:** Post-session therapist checklists

Did you manage to…	Body project mean (sd) Scale 1–5	Psycho-education mean (sd) Scale 1–5
Read the session protocol	3.01 (1.09)	3.47 (1.48)
Prepare the session with the co-therapist	2.45 (0.87)	2.77 (0.94)
Prepare hand-outs	4.46 (0.52)	4.27 (0.76)
Create a relaxed atmosphere during the session	4.26 (0.38)	4.26 (0.42)
Make the session fun/engaging	4.14 (0.43)	4.04 (0.32)
Activate all patients	4.37 (0.42)	4.18 (0.40)
Make eye contact with the patients	4.62 (0.33)	4.29 (0.47)
Use appropriate humor	4.28 (0.50)	3.40 (0-.39)
Smile	4.24 (0.66)	4.14 (0.44)
Be relaxed	4.13 (0.37)	4.25 (0.42)
Make empathic comments	4.25 (0.46)	4.15 (0.38)
Control patients who were too talkative	4.18 (0.26)	4.10 (0.41)
Involve quite patients	4.32 (0.27)	3.83 (0.48)
Stop disturbing behaviour	4.42 (0.54)	4.76 (0.27)

Table 4 shows scores on the evaluation questionnaires therapists completed at the end of the study, evaluating the treatment as well as the study procedures. On a 5-point rating scale, with 5 being the most positive score, therapists’ scores for the ‘treatment protocol’ ranged from 3.50 to 4.00 (length was scored 3.50, with 3.00 being the most optimal score), scores for ‘homework content’ were 3.50 (Psycho-education) and 4.00 (Body Project), scores for ‘quantity’ were 3.00 with 3.00 being the most optimal. ‘Number of sessions’ and ‘session length’ were also scored around optimal with 2.50 and 3.00. ‘Fit with the patient group’ scored almost optimal on all subscales, ranging from 4.00 to 5.00 on subscales (sessions, exercises, handouts, word choice with 5.00 being the most positive score and 2.50–3.00 for ‘balance between exercises and discussion’ with 3.00 being the most optimal score. The ‘general evaluation’ showed positive scores for treatment content (4.00–5.00), and 3.50 for the questionnaires. Therapists noted the Body Project group was a particularly good fit for patients who were a few months into treatment. ‘Study procedures’ (time consuming and bothersome) were rated from 3.00 to 4.00. With regard to the group, ‘active participation’ (4.50–5.00) and ‘atmosphere’ (4.00–4.50) were rated positively. ‘Motivation’ was rated between 3.00 and 4.00 with the note that patients first felt confronted in the Body Project group, as is to be expected in a therapy causing cognitive dissonance. Once involved, patients were motivated and participated actively. ‘Treatment effects’ ranged from 3.00 to 4.00 in the Psycho-education group and from 3.50 to 5.00 for the Body Project group, but with the remark that mixed groups of patients (a mixed group of patients with anorexia nervosa and bulimia nervosa) caused anxiety for some patients.

**Table 4:** Therapist evaluation questionnaire

	Questions	Body project Mean (sd) Scale 1–5	Psycho-education Mean (sd) Scale 1–5
		with 5 being the most optimal score for most questions, exceptions are explicitly mentioned below	
Treatment protocol	Clarity	4.00 (1.41)	3.50 (0.71)
	Length (1 = too short, 5 = too long)	3.50 (0.71)	3.50 (0.71)
	Handouts	4.00 (-)	4.00 (-)
Homework	Content	4.00 (-)	3.50 (0.71)
	Quantity (1 = too little, 5 = too much)	3.00 (-)	3.00 (-)
Sessions	Length (1 = too short, 5 = too long)	2.50 (0.71)	3.00 (-)
	Number of sessions (1 = too little, 5 = too many)	2.50 (0.71)	3.00 (-)
Fit with the patient group	Sessions	4.50 (0.71)	4.50 (0.71)
	Exercises	5.00 (-)	5.00 (-)
	Handouts	5.00 (-)	5.00 (-)
	Word choice	5.00 (-)	4.00 (-)
	Balance between exercises and discussion (1 = too many exercises, 5 = too much discussion)	2.50 (0.71)	3.00 (-)
General evaluation	Treatment content	5.00 (-)	4.00 (-)
	Preparation time	3.50 (0.71)	3.00 (-)
	Treatment timing (1 = too soon, 5 = too late)	3.00 (-)	4.00 (-)
	Questionnaires	3.50 (0.71)	3.50 (0.71)
Study procedures	Time consuming (5 = not at all)	4.00 (-)	3.00 (1.41)
	Bothersome (5 = not at all)	3.00 (-)	4.00 (-)
Group	Active participation	5.00 (-)	4.50 (0.71)
	Atmosphere	4.50 (-)	4.00 (-)
	Motivation	3.00 (1.41)	4.00 (-)
Treatment effects	Body dissatisfaction	4.00 (-)	3.00 (-)
	Eating disorder	3.50 (0.71)	3.50 (0.71)
	Self-esteem	5.00 (-)	3.00 (-)
	Negative effects (5 = not at all)	4.00 (1.41)	4.00 (-)
	Lasting effects	4.00 (-)	3.00 (-)
	Effectiveness for a mixed group of eating disorders	4.00 (-)	3.00 (-)
	Preference for a mixed group of eating disorders	4.00 (1.41)	3.00 (-)
Acceptability	Useful	5.00 (-)	5.00 (-)
	Fit with the patient group	5.00 (-)	5.00 (-)
	Downsides of treatment	5.00 (-)	4.50 (0.71)
	Bothersome for patients (5 = not at all)	3.00 (-)	3.00 (-)
	Bothersome for therapists	5.00 (-)	2.50 (0.71)
	Discomfort for patients	3.00 (-)	3.50 (0.71)
	Discomfort for therapists	5.00 (-)	4.00 (-)
	Difficulty with sharing therapists’ own experiences	4.50 (0.71)	3.00 (-)
	Motivation providing the treatment	5.00 (-)	4.50 (0.71)
	Willingness to provide the treatment in the future	5.00 (-)	5.00 (-)

The groups were rated highly acceptable, with maximum scores for ‘usefulness’, ‘fit with the patient group’, ‘being (not) bothersome for patients’ and ‘willingness to provide the treatment in the future’. According to the therapists, the Body Project group was a good fit for younger patients, although they needed more encouragement than adult patients did. Adult patients with bulimia nervosa, nearing the end of treatment seemed particularly able to distance themselves from the media. One negative remark stood out. Therapists mentioned that it was bothersome for both therapists (2.50–5.00) and patients (3.00) that both treatments had to be offered outside of regular treatment times (at the end of the day or even on a non-therapy day), which made scheduling of the treatments difficult.

#### Treatment directors and team

Treatment directors and the team evaluated the Body Project group and the Psycho-education group positively. Two main themes needing improvement became apparent in the treatment directors’ evaluation questionnaires. First, as both group treatments were offered outside of patients’ regular treatment schedule, this caused several issues. For example, it was difficult to recruit participants for the study, as patients had to attend six additional treatment sessions on top of their daycare treatment. For some patients this meant staying longer, for other patients this meant coming to the treatment centre during a non-treatment day. Treatment directors advised to incorporate the treatment groups into the treatment program for a future study. Second, and most importantly, treatment directors mentioned they saw improvements in their patients on eating disorder symptoms, body dissatisfaction, and self-esteem, although of course it remains unclear which effects were caused by the Body Project and Psycho-education groups and which effects were a result of standard treatment. All treatment directors answered they were willing to recruit patients in the future.

### Questionnaires

As the study was set up as a feasibility and acceptability pilot study, the study was underpowered to assess treatment effects. Preliminary analyses were carried out to explore effectiveness of the Body Project and Psycho-education groups. Table 5 shows means and standard deviations across all timepoints: pre-treatment, post-treatment, and at 3- and 6-months follow-up. Treatment as usual continued during the entire study period, including follow-up, and questionnaire scores show a decrease in symptoms across all times and across both groups.

**Table 5:** Questionnaire scores: means and standard deviations

Questionnaire	Body project *M* (*SD*) Pre	Psycho-education *M* (*SD*) Pre	Body project *M* (*SD*) Post	Psycho-education *M* (*SD*) Post	Body project *M* (*SD*) 3 month FU	Psycho-education *M* (*SD*) 3 month FU	Body project *M* (*SD*) 6 month FU	Psycho-education *M* (*SD*) 6 month FU
BAI – Anxiety symptoms	25.40 (11.30)	28.14 (9.09)	19.05 (9.74)	23.94 (11.32)	19.00 (12.17)	20.50 (13.43)	18.93 (15.09)	14.00 (6.16)
BDI—Depressive symptoms	31.68 (10.53)	36.75 (10.29)	30.09 (13.88)	33.33 (10.41)	23.86 (14.04)	29.50 (12.08)	23.97 (17.47)	19.67 (8.47)
PANAS – Negative affect	35.32 (9.57)	46.38 (8.24)	33.08 (9.46)	37.88 (12.36)	32.29 (11.56)	36.79 (13.91)	28.67 (13.45)	27.80 (10.16)
EDE-Q—Eating disorder symptoms	3.85 (1.19)	4.34 (0.95)	3.30 (1.52)	3.65 (1.25)	2.85 (1.34)	3.28 (1.16)	2.36 (1.76)	2.50 (1.02)
BAT – Body dissatisfaction	64.04 (14.96)	68.61 (12.17)	60.63 (19.40)	65.67 (17.12)	53.30 (16.90)	62.53 (19.18)	48.27 (18.72)	53.60 (14.81)
OBCS – Body objectification	123.86 (16.11)	128.82 (12.75)	111.79 (17.04)	121.31 (18.25)	114.48 (16.81)	120.60 (15.78)	105.80 (22.22)	116.00 (17.42)
Body Objectification—weight	7.00 (2.14)	6.65 (2.60)	5.63 (3.08)	6.56 (2.92)	5.10 (3.24)	5.67 (2.79)	5.00 (2.91)	5.20 (3.08)
SATAQ – thin-ideal internalization	110.30 (23.40)	113.78 (22.52)	106.21 (23.61)	108.31 (29.57)	101.33 (26.44)	105.80 (21.88)	93.54 (30.25)	107.22 (24.45)

The mixed model analyses showed that none of the group*time interaction effects were significant (BAI: *F*(1,1) = 0.820, *p* = 0.486; BDI: *F*(1,1) = 1.167, *p* = 0.326; PANAS: *F*(1,1) = 2.711, *p* = 0.049; EDE-Q: *F*(1,1) = 0.332, *p* = 0.802; BAT: *F*(1,1) = 0.116, *p* = 0.951; OBCS: *F*(1,1) = 0.476, *p* = 0.699; Body objectification: *F*(1,1) = 1.267, *p* = 0.290; SATAQ: *F*(1,1) = 0.279, *p* = 0.841). The interaction was therefore dropped from the model. The model with group and time as main effects showed a significant main effect of time for all questionnaires (BAI: *F*(1,1) = 38.258, *p* = 0.000; BDI: *F*(1,1) = 52.292, *p* = 0.000; PANAS: *F*(1,1) = 43.453, *p* = 0.000; EDE-Q: *F*(1,1) = 79.677, *p* = 0.000; BAT: *F*(1,1) = 38.134, *p* = 0.000; OBCS: *F*(1,1) = 25.026, *p* = 0.000; Body objectification: *F*(1,1) = 17.588, *p* = 0.000; SATAQ: *F*(1,1) = 13.951, *p* = 0.000), but no significant main effect of group (BAI: *F*(1,1) = 0.590, *p* = 0.446; BDI: *F*(1,1) = 1.287, *p* = 0.262; PANAS: *F*(1,1) = 6.950, *p* = 0.011; EDE-Q: *F*(1,1) = 1.936, *p* = 0.170; BAT: *F*(1,1) = 1.132, *p* = 0.292; OBCS: *F*(1,1) = 2.622, *p* = 0.111; Body objectification: *F*(1,1) = 0.175, *p* = 0.677; SATAQ: *F*(1,1) = 0.163, *p* = 0.688).

Table 6 shows that effect sizes for the time*group interaction were generally small. Negative affect (PANAS) showed a medium pre-post effect size, and a large pre-6 months follow-up effect size. The only other effect size reaching a medium size was Body objectification (OBCS) when comparing pre- to post-treatment.

**Table 6:** Within group effect sizes (pre- to post-treatment and pre-treatment to six months follow-up) and effect sizes for the time*group interaction (pre- to post-treatment)

Questionnaire	Cohen’s d pre-post Body Project	Psycho-education	Partial eta squared (time*group; t_1_ –t_3_)	Cohen’s d pre-6 months FU Body Project	Psycho-education	Partial eta squared (time*group; t_1_ –t_3_)
BAI – Anxiety symptoms	1.01	0.43	.001	0.54	1.77	0.047
BDI—Depressive symptoms	0.25	0.44	.005	0.94	1.84	.122
PANAS – Negative affect	0.29	0.68	.083	0.60	2.08	.115
EDE-Q—Eating disorder symptoms	0.80	0.89	.011	1.26	2.75	.002
BAT – Body dissatisfaction	0.25	0.27	.000	0.95	1.49	.028
OBCS – Body objectification	1.15	0.73	.091	0.91	1.46	.038
Body Objectification—weight	0.52	0.08	.007	0.72	0.68	.008
SATAQ – thin-ideal internalization	0.28	0.30	.009	0.81	0.22	.007

**Fig. 2:**
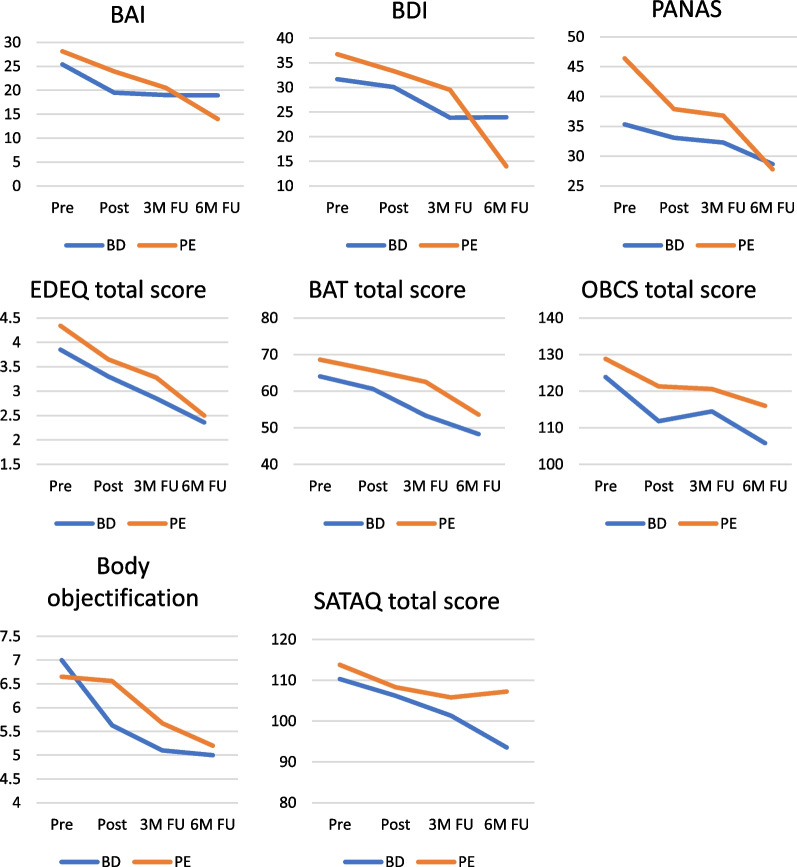
Pre-treatment, post-treatment and follow-up scores on all questionnaire total scores

Table 6 also shows within-group effect sizes for both treatments. Within-group effect sizes for the Psycho-education group were generally larger than those for the Body Project group, but symptoms of the patients in the Psycho-education group consistently started (and for some symptoms stayed) at a higher level, see also Fig. 2. Within-group effect sizes ranged from small to large. Medium to large effects for the Body Project group were found for Anxiety symptoms (BAI), Eating disorder symptoms (EDE-Q), and Body objectification (OBCS) when comparing pre- to post-treatment scores, and for all questionnaires when comparing pre-treatment scores to 6 months follow-up. For the Psycho-education group medium to large effects were found for Negative affect (PANAS), Eating disorder symptoms (EDE-Q), and Body objectification (OBCS) when comparing pre- to post-treatment scores, and for all questionnaire scores, with the exception of Thin-ideal internalization (SATAQ), when comparing pre-treatment scores to 6 months follow-up.

## Discussion

This study looked at feasibility and acceptability of the Body Project group, as an add-on to standard treatment, in a highly specialized eating disorder treatment centre in third-line mental health care. This is the first trial investigating the Body Project in a group of patients with severe eating disorders. We translated and adapted the Body Project protocol [32] for patients with severe eating disorders (see Appendix 1 for a complete overview). We chose to adapt the preventative Body Project instead of the Body Project Treatment [30]. The Body Project Treatment consists of eight sessions (instead of six as we have used in the present study) and Stice added exercises to address eating disorder symptoms next to body dissatisfaction and thin-ideal internalization. As our multimodal treatment program already includes several treatment modules targeting eating disorder symptoms, but lacks a treatment aimed at addressing the thin-ideal, we chose to adapt the original Body Project instead. Future trials in other treatment centres should carefully decide which version of the Body Project groups compliments their treatment best.

Patients with anorexia or bulimia nervosa either received six sessions of the Body Project group treatment or six sessions of a Psycho-education group treatment, both added to standard multimodal clinic or day-treatment. Feasibility and acceptability of both groups were evaluated from both the therapist and patient perspective and informed necessary modifications to the protocol. The study also looked at preliminary effectiveness of the Body Project group and the Psycho-education group on thin-ideal internalization, self-objectification, eating disorder symptoms, anxiety symptoms, depressive symptoms and negative affect. However, please note that results of the questionnaires should be interpreted with great caution, as analyses were of preliminary nature.

With regard to the study’s first aim, to test the feasibility and acceptability, the Body Project group as well as the Psycho-education group proved highly feasible and acceptable. Acceptability was reflected by a high attendance of sessions, and positive scores on questions evaluating treatment from both the patient and therapist perspective. Patients mentioned the exercises in the Body Project group were confronting and caused some discomfort. However, they also noted this was helpful. This uncomfortable feeling resulting from the exercises is in line with Festinger’s theory [9] on cognitive dissonance. Although the Psycho-education group was meant to be the control group in this study, patients also viewed this group as very helpful. Psycho-education is already part of our standard treatment, but it is not yet offered as a separate group treatment focusing on psycho-education alone. Rather, parts of psycho-education are added to different treatment modules. Patients commented that they liked the group format, the repetition of information and the ability to discuss the sometimes triggering information.

The qualitative feedback included several recommendations for future implementation and stress the need for further development of and modifications to the Body Project group before implementing it on a larger scale to treatment of patients with a severe eating disorder. First, patients commented they needed more than the current six sessions. Patients with eating disorders in a (highly) specialized treatment setting in third-line mental health care represent a different and more severe subset of eating disorder sufferers than previously investigated in trials testing the efficacy and effectiveness of the Body Project and the Body Project Treatment. The Body Project is a preventative treatment and trials therefore did not include patients with severe eating disorders. However, although targeting DSM 5 eating disorders, the Body Project Treatment trials [28–31, 33, 35, 36] still excluded patients that were not deemed appropriate for outpatient treatment, such as women with a BMI below 17, which makes sense, as the treatment is designed for outpatient treatment.

Second, our patient population does not only consist of adolescents, but also of adults and older adults. Moreover, some patients in our treatment centre have a longstanding eating disorder. Different versions of the manual may be needed in order to offer a more personalized treatment to the several subpopulations in (highly) specialized eating disorder care. An adolescent may benefit most from arguing the thin-beauty ideal, whereas an older woman needs to oppose to the pressure to look young and slim. Related to this, the treatment may benefit from homogeneous therapy groups with regard to age and with regard to eating disorder diagnosis. A single older woman may have trouble identifying herself with the rest of the group if the group consists of adolescents only (as was the case in one of our groups) and the same may hold for a single patient with bulimia nervosa in a group of patients with anorexia nervosa.

Also, additional psycho-education on cognitive dissonance may be needed to increase motivation, as cognitive dissonance feels quite uncomfortable, and patients may have the tendency to avoid this feeling. When the Body Project group is part of multidisciplinary treatment, as was the case in the present study, one may also think about integrating the Body Project group with for example psychomotor and drama therapy elements. Green et al. [11] and Rohde et al. [25] already added unique exercises to the standard treatment protocol, increasing engagement and adding focus on the costs of social comparison, producing large treatment effects.

Last, therapists noted the Body Project group seemed to be a particularly good fit for patients who were already a few months into treatment. This is in line with Fairburn’s CBT-E treatment protocol [7], where the body image module follows the core protocol where regular eating is established. When implementing the Body Project group as an add-on to a treatment similar to ours, timing is therefore an important factor to incorporate.

With regard to the study’s second aim, to assess preliminary effectiveness of the Body Project group and Psycho-education group, effect sizes varied and were generally larger for the Psycho-education group than for the Body Project group. However, symptoms of the patients in the Psycho-education group consistently started (and for some symptoms stayed) at a higher level. Nevertheless, it is impossible to disentangle the effects that resulted from the Body Project or Psycho-education group with effects that resulted from standard treatment. The inability to disentangle treatment effects is the largest limitation of our study. A second important limitation is the selection bias; 59% of the eligible patients declined participation (before randomization), mostly due to time logistic reasons (treatment offered outside therapy hours/days). Third, we excluded males, as the vast majority of our treatment population is female. Although the Body Project treatment has been provided to mixed-gender groups (see for example [15], it has not been thoroughly investigated yet, and evidence suggests treatment effects are bigger in single-sex groups [29, 31, 36]. Last, this study was set up as a feasibility and acceptability pilot study, and effectiveness was only assessed preliminary. Our study was therefore underpowered to assess treatment affects, and we could not assess moderating effects of eating disorder diagnosis, age and duration of the eating disorder.

It will be important to conduct fully powered efficacy and effectiveness trials of the Body Project group in a group of patients seeking treatment in (highly) specialized eating disorder services in third-line mental health care. Before conducting a large-scale RCT more intervention development and preliminary research is necessary, however, for the use of the Body Project intervention in (highly) specialized eating disorder services. Future research may for example focus on treatment moderators, such as for which clients the Body Project group is best suited (e.g., diagnosis, age, duration of the eating disorder)and when in the course of treatment the group should be provided for most optimal results. Results also showed the benefits of implementing a structured Psycho-education group where patients can discuss triggering information.

In conclusion, the Body Project group and the Psycho-education group, as well as study procedures, were highly acceptable and feasibly from the patients, therapists and treatment directors perspective. Preliminary analyses to establish effectiveness are difficult to disentangle from standard treatment effects. However, also based on patients’ written feedback, effects seemed positive. Future implementation of and future studies into the Body Project group may profit from the feedback generated by our study, including increasing the number of treatment sessions, creating homogeneous therapy groups with regard to age and eating disorder diagnosis, and optimizing timing of the treatment, especially when considering patients with severe underweight.

## Data Availability

The datasets used and/or analyzed during the current study are available from the corresponding author on reasonable request.

## Appendix 1

**Table:**

Session	Original program	Changes
General changes	Group website where participants interact outside the group	Patients are not allowed to have contact outside of treatment
	Recordings of several exercises	We made no recordings of the several exercises (e.g., reading the letter from session 2 or the body activism from session 5), as patients were not allowed to share the content outside of treatment
1	Letter to a younger girl: Costs of the thin-ideal	We added a YouTube video about Photoshopping
	Self-affirmation exercise	The original protocol asks to wear as little clothing as possible during this exercise. As wearing little clothing was considered too triggering, we omitted this instruction
2	Role plays to discourage the pursuit of the thin-ideal/verbal challenges	No changes
3	Quick comebacks	No changes, however, we changed some examples to Dutch culture (our patients for example do not know who Jennifer Lopez is)
	Behavioural challenge	No changes
4	Fat talk role play	Therapists first role-played together as an example
	Media misrepresentation news flash	No changes
	Group body activism	As patients were not allowed to have contact outside of treatment, we allowed them to stay an additional 30 min after the group ended to come up with a group activity
5	Group body activism at your school	Most patients, at least currently, do not attend school, and outside of treatment they are not allowed to have contact. We changed this exercise to (individual) body activism in your own social environment
	Future pressures to be thin	We slightly adapted this to future consequences of eating disorders
	Letter to a young girl	No changes
6	Self-affirmation exercise	Patients picked three ideas instead of one to practice with

## Appendix 2 Correlations between all questionnaire (sub)scales.

**Table:**

	1	2	3	4	5	6	7	8	9	10	11	12	13	14	15	16	17	18	19	20	21	22
**1** BAI	–	0.69	0.52	0.46	0.33	0.43	0.28	0.45	0.46	0.38	0.52	0.24	0.20	− 0.01	0.35	0.05	0.24	0.00	− 0.04	0.06	0.11	− 0.05
**2** BDI		–	0.57	0.65	0.44	0.58	0.52	0.56	0.63	0.50	0.62	0.38	0.47	0.24	0.49	0.24	0.28	0.16	0.09	0.03	0.29	0.13
**3** PANAS			–	0.42	0.29	0.40	0.35	0.41	0.39	0.36	0.32	0.22	0.38	0.21	0.43	0.05	0.10	0.20	0.14	0.16	0.19	0.20
**4** EDEQ				–	0.84	0.87	0.88	0.84	0.77	0.70	0.67	0.47	0.59	0.34	0.63	0.29	0.54	0.21	0.12	0.19	0.22	0.20
**5** EDEQ restraint					–	0.68	0.64	0.55	0.56	0.54	0.49	0.35	0.41	0.25	0.44	0.17	0.36	0.15	0.12	0.27	0.03	0.13
**6** EDEQ eating						–	0.68	0.66	0.53	0.47	0.58	0.21	0.36	0.06	0.54	0.28	0.32	0.00	− 0.08	0.13	0.07	− 0.01
**7** EDEQ weight							–	0.74	0.66	0.68	0.53	0.42	0.52	0.25	0.60	0.23	0.52	0.23	0.12	0.23	0.21	0.23
**8** EDEQ shape								–	0.74	0.69	0.52	0.57	0.63	0.46	0.63	0.23	0.45	0.35	0.25	0.12	0.41	0.34
**9** BAT									–	0.90	0.76	0.71	0.68	0.41	0.72	0.26	0.45	0.42	0.30	0.24	0.44	0.42
**10** BAT size										–	0.54	0.64	0.61	0.36	0.69	0.16	0.44	0.39	0.32	0.15	0.35	0.39
**11** BAT familiarity											–	0.23	0.38	− 0.04	0.54	0.36	0.37	0.10	− 0.07	0.35	0.18	0.07
**12** BAT dissatisfaction												–	0.52	0.58	0.47	− 0.06	0.25	0.60	0.57	0.09	0.57	0.55
**13** OBCS													-	0.70	0.81	0.57	0.44	0.50	0.42	0.04	0.53	0.49
**14** OBCS surveillance														–	0.34	0.02	0.30	0.63	0.64	0.03	0.53	0.63
**15** OBCS shame															–	0.34	0.35	0.33	0.22	0.17	0.38	0.30
**16** OBCS control																–	0.34	− 0.04	− 0.10	− 0.14	0.15	− 0.04
**17** Weight																	–	0.13	0.11	− 0.04	0.15	0.13
**18** SATAQ																		–	0.94	0.34	0.86	0.94
**19** SATAQ general																			–	0.18	0.78	0.85
**20** SATAQ athlete																				–	0.08	0.23
**21** SATAQ pressure																					–	0.75
**22** SATAQ information																						–
